# Food venue choice, consumer food environment, but not food venue availability within daily travel patterns are associated with dietary intake among adults, Lexington Kentucky 2011

**DOI:** 10.1186/1475-2891-12-17

**Published:** 2013-01-29

**Authors:** Alison Gustafson, Jay W Christian, Sarah Lewis, Kate Moore, Stephanie Jilcott

**Affiliations:** 1 University of Kentucky, Department of Nutrition and Food Science , Lexington, KY 40506, USA; 2 East Carolina University Brody School of Medicine, Department of Public Health, Greenville, NC, USA; 3 University of Kentucky, Department Geograpghy, Lexington, KY, 40506, USA

**Keywords:** Food store availability, Food environment, Dietary habits

## Abstract

**Objective:**

The retail food environment may be one important determinant of dietary intake. However, limited research focuses on individuals’ food shopping behavior and activity within the retail food environment. This study’s aims were to determine the association between six various dietary indicators and 1) food venue availability; 2) food venue choice and frequency; and 3) availability of healthy food within food venue.

**Methods:**

In Fall, 2011, a cross-sectional survey was conducted among adults (n=121) age 18 years and over in Lexington, Kentucky. Participants wore a global position system (GPS) data logger for 3-days (2 weekdays and 1 weekend day) to track their daily activity space, which was used to assess food activity space. They completed a survey to assess demographics, food shopping behaviors, and dietary outcomes. Food store audits were conducted using the Nutrition Environment Measurement Survey-Store Rudd (NEMS-S) in stores where respondents reported purchasing food (n=22). Multivariate logistic regression was used to examine associations between six dietary variables with food venue availability within activity space; food venue choice; frequency of shopping; and availability of food within food venue.

**Results:**

1) *Food venue availability within activity space* – no significant associations. 2) *Food Venue Choice* – Shopping at farmers’ markets or specialty grocery stores reported higher odds of consuming fruits and vegetables (OR 1.60 95% CI [1.21, 2.79]). *Frequency of shopping* - Shopping at a farmers’ markets and specialty stores at least once a week reported higher odds of consumption of fruits and vegetables (OR 1.55 95% CI [1.08, 2.23]). Yet, shopping frequently at a super market had higher odds of consuming sugar-sweetened beverages (OR 1.39 95% CI [1.03, 1.86]). 3) *Availability of food within store* – those who shop in supermarkets with high availability of healthy food has lower odds of consuming sugar-sweetened beverages (OR 0.65 95% CI [0.14, 0.83]).

**Conclusion:**

Interventions aimed at improving fruit and vegetable intake need to consider where individuals’ purchase food and the availability within stores as a behavioral and environmental strategy.

## Introduction

In the past several years, researchers have focused on the retail food environment as a determinant of dietary intake and weight status. To date, there appears to be no definitive conclusion of how the retail food environment is associated with dietary intake or patterns [1-4]. Yet, conceptually, it is clear that the food environment plays a role in influencing dietary choices [5]. Both cross-sectional and longitudinal studies, have found varied results: some studies conducted in urban settings have found associations between neighborhoods with fewer supermarkets and higher rates of obesity and poor dietary habits [6-12]. Additionally, other studies measuring the retail food environment (number of healthy stores relative to unhealthy stores) have found that those counties with more healthy food stores have lower average body mass index of residents [13,14]. Conversely, others have found that living closer to a supermarket is not associated with fruit and vegetable consumption [15,16]. Yet, given the complex interdependent relationship between the individual and the food environment, new strategies are needed to further disentangle individual’s interactions with the retail food environment and dietary outcomes.

Recently researchers have tracked daily movement patterns as a way to understand how individuals behave within their neighborhoods [17]. Through the use of global positioning system (GPS) data loggers the neighborhood can be organically defined through the individuals’ daily living habits. Neighborhood, in this case, is defined by the individuals’ daily travel throughout and not through the use of geographically derived boundaries such as census tracts or zip codes. The shape of the neighborhood is captured by daily travel patterns; however this definition still misses how a neighborhood might influence travel patterns and routes. The use of these new technologies allows for an improved measurement of the neighborhood but still does not capture how the neighborhood food environment influences choice of food venue or availability of food inside the venue and subsequent dietary intake. Two recent studies utilizing GPS technologies indicated that people traveling within a high density of fast-food restaurants had higher odds of being overweight [18] and consuming more saturated fat [19]. While understanding travel patterns and how food venue exposure within those travel patterns is needed, food shopping choices or behaviors which may influence travel patterns is equally relevant. Recent results find that few individuals choose to shop in the store that is nearest to them [20,21]. Thus proximity of store to home may not be as relevant as other determinants of why individuals choose one store type over another. Findings related to food store choice have indicated that those who shop at a grocery store in a disadvantaged neighborhood report a higher body mass index (BMI) relative to those who shop at a grocery store in a higher income neighborhood [22,23]. Yet, among those who “choose” to shop at store in a disadvantaged neighborhood this may be more of a function of socio-economic status, transportation, and a host of other proximal and distal determinants of dietary intake which influence choice.

To adequately capture the interdependent nature between the individual and their neighborhood both choice of food venue and availability of healthy food within venue are necessary. To date studies have found that availability of healthy food within stores may or may not be associated with dietary intake and body mass index [11,24,25]. The conflicting results may be more a reflection of the methods used, sample population, and also the reality that supermarkets sell more produce but they also sell more unhealthy items, such as snack foods, cake, cookies, and other processed food at the same time [26]. Individual’s when food shopping are faced with the decision to purchase a healthy food item at the same time they are faced with the decision to buy an unhealthy food item. Impulsivity, marketing, place, promotion, price, family and culture all influence the decisional balance between a healthy item and an unhealthy item [27-29].

By assessing the travel patterns through the GPS data loggers, where primary and secondary food shopping takes place, and availability of healthy food within food venues a more nuanced understanding of the role the retail food environment plays in individual’s choices can be gleaned. The aims of this study were to examine associations between various dietary indicators and 1) food venue availability within daily activity space; 2) food venue choice and frequency of shopping (supermarket, supercenter, specialty grocer, farmers’ market) and 3) healthy food availability within the food venue. The primary hypothesis was that those who travel within an area that has more healthy food venues relative to less healthy food venues will report higher intake of healthy foods. Additionally, those who shop in healthy food venues (defined as super markets, farmers’ markets, specialty markets) will report greater healthy food consumption. Lastly, the third hypothesis was that those who shop in stores with high availability of healthy food would report greater healthy food consumption.

## Methods

### Study setting and design

Lexington, KY (population=295,803) is 283 square miles with approximately 1,000 people per square mile, is a small urban city surrounded by rural communities. The racial and ethnic composition of Lexington, KY is 76% White, 15% Black, 7% Hispanic or Latino, and 2% Asian based on the United States Census Bureau 2010 [30]. This cross-sectional study occurred June – September 2011 and examined food activity space using Global Positioning System (GPS) devices (described below), and a survey to assess food shopping behaviors and dietary intake.

### Participant recruitment and survey administration

Lexington residents were recruited to participate in a study assessing “activity space,” or their daily patterns of travel, using GPS data loggers. Participant recruitment consisted of mailed flyers describing the research and contact information. Participants were eligible if they met the following criteria: 1) have lived in Lexington for at least one year; 2) indicated that they were not moving in the next year; 3) 18 years of age or older; 4) and had no reported health condition which would preclude the participant from their daily living activities. A list of 1400 household or apartment building addresses were obtained from a statewide survey on cancer control and prevention habits conducted through the Markey Cancer Center at the University of Kentucky. The participants in the survey were not currently diagnosed with cancer but may have undiagnosed chronic disease. Approximately 1400 households received over 3100 flyers with low response households (n=300) receiving an additional flyer inviting them to participate. Each household received at least two flyers on separate occasions several weeks apart. For those households that were categorized as low-response (no response was received from research staff within two weeks after flyer was mailed) a third flyer was mailed to that household. A total of 153 persons responded but only 121 were eligible to participate based on the requirements (153/1400 = response rate of 11%). Of the 32 respondents that were ineligible, all but two had not lived in Lexington for less than one year, and the other two did not agree to wear the GPS data logger for one week (two weekdays and one weekend day). The University of Kentucky Institutional Review Board approved the study, and participants signed informed consent upon enrollment into the study.

The survey was initially administered via the telephone to all study participants by a graduate assistant. The graduate assistant was trained by the University of Kentucky Survey Research Center (SRC) in the procedures for collecting data via the telephone. The training within the SRC consists of internal review board training, sensitivity training, as well as programmatic training. This study provided salary support for a graduate assistant staffed at SRC to conduct the telephone survey. The survey included questions regarding demographics (age, race, gender, marital status, length of residence in Lexington and in current residence, education level, annual income, employment status, weight, height, and automobile ownership) and food shopping behavior, which were adapted from previous surveys [8,15,24,31,32]. The validated National Health and Nutrition Examination Survey (NHANES) 2009–2010 dietary screener [33] was administered to assess dietary intake variables. The survey took approximately 40 minutes (mean 38 minutes SD 1.52 with a range of 32–44).

### Independent variables

#### Food venue availability within daily activity space

To capture what type of stores were within an individuals’ daily activity space three steps were taken. Step one consisted of collecting food venue addresses and verifying the location. To categorize each food venue, a list of Lexington food venues was obtained from InfoUSA in June 2011. To verify that stores were open and located ground-truthing was conducted [31]. Such that, once the daily activity space was categorized the food stores that were within those spaces were verified to be open and located by driving to each store and comparing the list of stores on InfoUSA with what was found. All stores were located and open within the activity spaces of participants. Farmers’ markets and produce stands were obtained from the Fayette County Health Department. Food venue types were categorized based on name recognition and based on North American Industry Classification System (NAICS) Codes. The categories reflected supercenters (I.e. Super Walmart), supermarket/grocery stores (I.e. Kroger), specialty stores (I.e. Whole Foods) convenience stores (I.e. Seven Eleven), and gas stations with convenience stores, respectively. Dollar stores were not included in this analysis since the RFEI (described below) has not used this type of food venue before. However, future studies need to consider the use of this non-traditional food venue as a substantial source of calories and food purchases [34].

Step two consisted of identifying the food venues available within the individuals’ daily activity space. Food venue availability was measured using data from the GPS data loggers to count how many food venues were within the individuals’ daily activity space. For example, a person traveling from their home to work may pass one convenience store, two supermarkets, and one supercenter. Therefore their food venue availability consists of one convenience store, two supermarkets, and one supercenter.

Step three was to determine the healthfulness of an individual’s daily activity space. To measure the healthfulness of an individual’s daily activity space the retail food environment index (RFEI) was used [35,36]. The RFEI is a ratio of healthy relative to unhealthy food venues: Supermarkets/grocery stores, farmers’ markets, and produce stands were considered healthy venues, relative to supercenters, convenience stores, fast-food restaurants, and gas stations with convenience stores, or less healthy venues. The RFEI has been used previously to find a link between diabetes and obesity such that, a higher RFEI was associated with a higher prevalence of obesity and diabetes in low-income neighborhoods [37]. In this study supercenter was used in the numerator and denominator but point estimates did not change significantly, therefore it was retained in the numerator based on previous studies linking proximity to supercenters and high body mass index [38]. The RFEI was derived by adding up the number of food venues within a ½ mile buffer around the individuals’ daily activity space. The numerator is the number of healthy stores within the activity space while the denominator is the number of unhealthy stores within the activity space. In the ratio measure used here, a higher score indicated a travel pattern where individual’s encountered more stores selling fruits, vegetables, and other nutrient dense items. A lower score indicated a travel pattern where individual’s encountered more stores selling snack items, processed foods, and high calorie items.

#### Daily activity space

GPS data loggers can be used to capture daily activity space, or where an individual travels on a daily basis [17]. GPS data loggers allow researchers to create participant-defined neighborhoods, versus reliance on neighborhoods defined by administrative boundaries (e.g., Census tracts) or investigator-defined Geographic Information System (GIS) buffers [39,40]. In the current study, individuals’ daily activity spaces were derived by having participants wear a GPS data logger (Qstarz BT-1000XT Travel Recorder) for three days (two weekdays and one weekend day) during a seven day week to record all locations at a given time. The daily activity space is the actual path that individuals travel captured by the GPS data logger. For each interval, a map was created reflecting the participants’ daily activity space. Within the daily activity space, ArcGIS map software tools identified all food venue locations within 2640 feet (1/2 mile) of each participant’s three-day daily activity space. The resulting data set contained the participants’ counts of each food venue type (described below) within the daily activity space.

#### Food shopping behaviors

Participants were asked to provide name and location of up to three food venues (where they purchase food during the week or month. Supermarkets and grocery stores were categorized together as no participants reported shopping at a smaller grocery store. Specialty grocery stores (i.e. Whole Foods) were further categorized based on number of cash registers (five or less) and store name recognition. As those who shopped at farmers’ market also reported shopping at specialty stores (r=0.9), specialty stores and farmers’ markets were grouped together.

Frequency of shopping at each store type was captured by asking how often in a week or month do you shop at each store type (super market, convenience, farmers’ market, specialty market). Frequency was categorized as zero or never for shopping at store type compared to shopping at store type at least once a week based on the distribution of the data.

#### Consumer food environment

Twenty-two food venues were reported by participants as the primary food store for shopping. Similar food outlets were frequented by several individuals and although the sample was n=121 adults, only 22 different food outlets were listed. As indicated in Table 1, 76% reported shopping at a supermarket, 11% at supercenter, and 10% at specialty market for their primary food store and as such food audits were not conducted at farmers’ markets or convenience stores. Food store audits using a modified version of the Nutrition Environment Measurement Survey-Stores [41] (NEMS-S Rudd) [42] was used to assess the consumer food environment. NEMS-S Rudd is used to assess the overall availability of healthy food items relative to regular food items within a food venue. Based on the NEMS-S protocol, availability, price, and quality of food were collected for 15 food categories (fruit, vegetables, milk, cheese, meat, baked goods, chips, beverages, canned items, cereal, desserts, prepared food items, snack foods, frozen meals, and beans) and 55 unique food items were assessed. Availability and price per unit were recorded on the NEMS-S audit sheet. Price was collected by capturing the price per unit or ounce for each food item of the lowest priced item. Price was given a score based on if the healthy item were equal or lesser in value relative to the less healthy item. Therefore the comparison was between products within the same category. Price was collected for example on the lowest priced milk of the same brand across different sizes. In regards to produce price was collected for example on cantaloupe as either per pound or item depending on what was advertised. Thus, price was not all converted to ounces but was compared across categories of food. Quality was measured per NEMS-S protocol (NEMS-S) with produce items receiving a one if deemed acceptable for purchase and consumption (no bruises, dents, molding) or a zero if not acceptable for purchase and consumption. The modifications consisted of adding two store prepared meal options (Rotisserie and fried chicken meals, mixed green salad and mayonnaise based vegetable salad) and three snack food items (low-fat and regular potato chips, regular and reduced-fat cookies, ice-cream and reduced-fat ice cream) based on trends in consumption [43] and previous research regarding the need to assess availability of ready-made meal and snack options in such stores [11,44].

**Table 1:** Demographics Lexington, Kentucky 2011

**n=121**	**Percent**	**Mean**	**SD (+/−)**
**Age**		42	11.88
**Years in Lexington**		21	14.91
**Years in Neighborhood**		10	11.25
**Gender**			
Male	42%		
Female	58%		
**Race**			
White	94%		
African American	2%		
Other	4%		
**Marital Status**			
Single-never married	22%		
Married	50%		
Living with significant other	9%		
Divorced or separated	17%		
Widowed	2%		
**Education**			
Did not finish High school	<1%		
High School degree (includes GED)	7%		
Some college	12%		
Associates Degree	2%		
Bachelor’s Degree	35%		
Post-Secondary Degree	43%		
**Annual Income**			
$15,000 or less	8%		
$15,001-$25,000	9%		
$25,001-$50,000	21%		
$50,001-$75,000	23%		
$75,001-$100,000	20%		
More than $100,000	17%		
**Employment Status**			
Employed full-time	70%		
Employed part-time	21%		
Homemaker	2%		
Disabled	2%		
Retired	2%		
Unemployed	3%		
**Automobile ownership**			
Yes	95%		
No	5%		
**BMI**			
Underweight	4%		
Normal weight	55%		
Overweight	27%		
Obese	14%		

The store audits were conducted Monday-Friday between nine am - five pm by graduate students trained in the NEMS-S protocol. NEMS-S online training certification [41] was obtained by the principal investigator and subsequent trainings were given to the graduate students conducting the assessments. Two trained graduate assistants conducted all the NEMS-S audits for all food venues participants listed that were the primary food store for shopping. The graduate students conducted the audits within the same week but on different days of the week. All venues listed were located and open. All researchers reviewed audit sheets for completeness and accuracy. A kappa coefficient was calculated for each food venue to test inter-rater reliability. The kappa threshold, k ≥ 0.60, was used to determine if re-audits were necessary [45]. If a kappa coefficient was lower than 0.60, the establishment was re-audited. Kappa coefficients ranged from 0.85-0.95. Since no store had a kappa equal or lower than 0.60 no store was re-audited.

The NEMS-S scoring system provides a composite “total” score, and sub-scores for healthy food availability, pricing, and quality [41]. A tally sheet was used to determine if the food item was available at the time of the audit. The food item received a one if it was available and a zero if it was not available. The scoring system for price varies depending on the food item which is based on the protocol established by the Glanz, et al. [41]. Quality received a one if it was deemed acceptable for purchase and consumption (no bruises, dents, molding) or a zero if it was not acceptable for purchase and consumption. The total score ranged from zero-80, the availability sub-score ranged from zero-55, price sub-score ranged from zero-15, and quality sub-score ranged from zero-10. Higher NEMS-S scores indicated stores with better availability, price and quality of healthy foods. The total NEMS-S score range from all stores was 32–67, availability ranged from 26–47, price two-nine, and quality was six with no variance between stores. A store with a higher score would indicate that there were more healthy items at lower cost and of higher quality. For each store type where NEMS-S was conducted (supermarkets, supercenters, and specialty stores) different cut-points were created to define a store as “healthy” and “unhealthy” defined as high availability + low prices + high quality as “healthy” and low availability + high prices + low quality as “unhealthy”. Supermarket and specialty market cut point was 56 based on median score among those who shop at supermarkets for their primary store. Supercenter cut point was 57 based on median score among those who shop at supercenters for their primary food store.

### Dependent variables

#### Dietary variables

Dietary questions were from the validated National Health and Nutrition Examination Survey (NHANES) 2009–2010 dietary screener [33,46]. The Dietary Screener Questionnaire is composed of 26 questions that ask about the frequency of consumption in the past month of selected foods and drinks to capture intakes of fruits and vegetables, dairy/calcium, whole grains/fiber, added sugars, red meat, and processed meat. The following dietary variables were used to examine associations between intake of specific foods and availability, price, and quality of foods as determined via the NEMS-S food store audits: *Fruit and vegetable, milk, red meat, high fiber cereal, regular soda pop, sweetened fruit drinks, sweet rolls, muffins, cookies, cake, pie, doughnuts, and ice cream.* The following dichotomous food categories were used for all dietary variables based on distribution of the data. The data were skewed and not evenly distributed amongst the various categories and thus the median value was used for each food category. (1) Fruit and vegetable: less than two times per day and two times or more per day; (2) Milk: less than once per day and once or per day; (3) Red meat: less than two times per week and two times or more per week; (4) High fiber cereal: less than once per day and once or more per day; (5) Sweetened beverages (includes regular soda pop and sweetened beverages): never and at least once per week; (6) Baked goods and sweets (includes sweet rolls, muffins, cookies, cake, doughnuts, pie, and ice cream): less than five times per week and five times or more per week.

Fruit consumption was derived by asking participants “How often (times per month) do you eat fruit? Include fresh, frozen or canned fruit. Do not include juices. Do not include dried fruits.” Vegetable consumption was derived by asking participants “How often (times per month) do you eat a green leafy or lettuce salad, with or without other vegetables? Include: Spinach salads.” “Not including what you just told me about (lettuce salads, potatoes, cooked dried beans), how often do you eat other vegetables? Examples of other vegetables include: carrots, corn, cabbage, bean sprouts, collard greens, and broccoli. All other dietary questions were asked in the following format: “How often (times per month) do you consume _____ (milk, red meat [pork, beef, ham, or sausage], high fiber cereal [cereal name was given and fiber content was assessed with diet database to determine grams of fiber], baked good and sweets [doughnuts, pastries, chocolate, ice cream, cookies, cakes, pie, or brownies] and sweetened beverages [regular soda or pop but not diet soda, sweetened fruit drinks, sports or energy drinks, such as KoolAid, lemonade, HiC, cranberry drink, Gatorade, Red Bull or Vitamin Water, and coffee or tea that had sugar or honey added to it]). Participants could answer per day, week, or month. The variables were recalculated to assess consumption during a day or week depending on distribution of the data.

### Statistical analysis

Descriptive statistics (percentages, means, and standard deviations) were used to describe the study participants. To examine differences between NEMS-S scores and food venue types, t-tests were used. Logistic regression was used to examine associations between dietary intake, food venue type, and healthy food availability (NEMS-S score for availability), adjusting for age, race, gender, employment, daily activity space (1/2 mile pattern), and RFEI score. All models contained a cluster command for census tract since individuals were nested within the same census tracts precluding assumptions of independence of observations.

For each dietary variable, four logistic regression models were constructed. Model one assessed the odds of consumption of each dietary variable given a daily activity space that has a high retail food environment index (RFEI). Model two assessed the odds of consumption of each dietary variable given the type of food shopping venue. Model three assessed the odds of consumption of each dietary variable given the frequency of shopping at each food venue. Model four assessed the odds of consumption of each dietary variable given shopping in stores with availability of healthy food items within stores. Likelihood ratio tests were conducted to assure logistic regression analyses was appropriate for all models. All analyses were conducted using Stata (version 11.0, 2009, College Station, TX) [47].

## Results

The study sample in analysis was n=121. Participants had a mean age of 42 years and had lived in Lexington a mean of 21 years (Table 1). The sample was of a high socio-economic status with 35% of the sample having a college degree, and 60% earning over $50,000 per year. On average individual’s consumed a little over 7 fruits and 8 vegetables per day. 76% shopped in a supermarket as their primary food store and shopped at that store a little over 2 times per week. While wearing the GPS data logger 75% of the individual’s reported food shopping at their primary or secondary food store. On a range between 32–67 points out of a total of 80 points, the average NEMS-S score was 56.45. (Table 2) .

**Table 2:** Dietary and food shopping behaviors among study sample, Lexington, KY 2011

	**Percent**	**Mean**	**SD (+/−)**
**Diet (Times/week)**			
Fruit		7.67	6.31
Vegetables		8.22	6.21
Green leafy vegetables		3.84	2.71
Cereal		2.62	3.72
Red Meat		3.31	3.81
Milk		3.27	3.86
Soda		1.22	2.96
Sweetened Beverages		1.06	3.25
Baked sweets and desserts		5.55	1.51
**Frequency of store shopping (per week)**			
Supermarket		2.27	1.67
Supercenter		0.2	0.56
Specialty Market & Farmer's Market		0.27	0.54
Convenience Stores		0.62	1.43
**Food Store Scores**			
Mean Total Score (range 32–67)		56.45	3.51
Mean Availability Score (range 26–47)		45.47	2.41
Mean Quality Score (all stores received 6)		6	
Mean Price Score (range 2–9)		5	2.49
**Type of Store for food shopping (Primary)**			
Supermarket	76%		
Supercenter	11%		
Specialty Markets (i.e. Whole Foods)	10%		
Farmer's Market	0%		
Other (Amazon, Small grocery store)	3%		
**Type of Store for food shopping (Secondary)**			
Supermarket	41%		
Supercenter	21%		
Specialty Market (i.e. Whole Foods)	27%		
Farmer's Market	5%		
Supermarket discount (Aldi's)	5%		
Fruit and Vegetable stand	1%		
**Pattern of Food Purchases (Food venues where individuals reported purchasing food from when wearing GPS data logger)**			
Supermarket	75%		
Supercenter	<1%		
Convenience Stores	<1%		
Coffee Shop	30%		
Specialty Market	5%		
Fast-Food Restaurant	23%		
Ice-Cream Shop	4%		
No food was purchased	5%		

In the model assessing the availability of healthy food venues within the individual’s travel pattern and their dietary intake, no significant results were found (Table 3). There were no greater or lesser odds of consuming fruits and vegetables, milk, red meat, high fiber cereal, or sugar-sweetened beverages if a person traveled within a healthier retail food environment compared to traveling within a less healthy retail food environment.

**Table 3:** Retail Food Environment Index Score within travel space and the association with dietary intake, Lexington, KY 2011

	**Fruit & Vegetable**	**Sweetened Beverages**	**Red Meat**	**Milk**	**Baked goods and sweets**	**Cereal**
**Retail Food Environment Index (RFEI) Score within 1/2 mile Activity Space**	0.91 (0.52, 1.60)	0.66 (0.36, 1.24)	1.04 (0.59, 1.83)	0.84 (0.46, 1.57)	0.82 (0.47, 1.41)	1.24 (0.70, 2.20)

All models adjusted for education, income, race, employment, age, and gender.

Sweetened beverage is never consuming a sweetened beverage during the week.

Fruit &veg recommendation ref is eating vegetable, green leafy and fruit less than 2 times per day.

Baked goods and sweets is less than 5 times per week.

Milk is less than once per day.

Cereal is less than once per day.

Red meat is less than 2 times per week.

RFEI retained linear shape.

In models examining the association between dietary intake and type of food shopping venue (Table 4), shopping at a specialty or farmers’ markets was associated with higher odds of consuming fruits and vegetables at least two times per day compared to those who never shop at specialty store or farmers’ market. Additionally, those who shop supercenters had lower odds of consuming sweetened beverages compared to those who do not shop at supercenters. No significant associations were found for any of the other dietary variables.

**Table 4:** Type of food store for food shopping, frequency, and the odds of dietary intake, Lexington, KY 2011

	**Fruit & Vegetable**	**Sweetened Beverages (including regular soda)**	**Red Meat**	**Milk**	**Baked Goods and Sweets**	**Cereal**
**Super Markets**	1.14 (0.52, 2.53)	1.35 (0.57, 3.20)	0.85 (0.39, 1.91)	0.81 (0.34, 1.92)	0.74 (0.33, 1.57)	1.08 (0.49, 2.41)
**Super Centers and Warehouse Clubs**	1.21 (0.41, 3.53)	0.27 (0.09, 0.83)*	3.27 (0.98, 10.87)	0.30 (0.07, 1.49)	1.60 (0.54, 4.67)	0.67 (0.23, 2.01)
**Specialty Stores and Farmers' Markets**	1.60 (1.21, 2.79)*	1.19 (0.65, 2.16)	0.96 (0.54, 1.67)	1.24 (0.68, 2.29)	0.69 (0.40, 1.20)	0.74 (0.42, 1.28)
**Convenience Stores**	0.94 (0.41, 2.13)	1.37 (0.56, 3.35)	1.28 (0.56, 2.93)	1.34 (0.54, 3.33)	1.34 (0.60, 3.01)	1.31 (0.57, 3.04)
	**Frequency of Shopping for each store type (Odds Ratio and 95% Confidence Interval)**					
	**Fruit & Vegetable**	**Sweetened Beverages (including regular soda)**	**Red Meat**	**Milk**	**Baked Goods and Sweets**	**Cereal**
**Super Markets**	0.89 (0.37, 2.17)	1.39 (1.03, 1.86)*	0.93 (0.37, 2.31)	0.70 (0.27, 1.78)	0.70 (0.30, 1.63)	1.19 (0.49, 2.89)
**Supercenters and Warehouse clubs**	0.56 (0.13, 2.45)	0.37 (0.17, 0.78)*	4.09 (0.90, 15.55)	0.42 (0.08, 2.39)	0.62 (0.22, 1.72)	0.47 (0.12, 1.74)
**Specialty Stores and Farmer's Market**	1.55 (1.08, 2.23)*	1.21 (0.46, 3.20)	1.58 (0.65, 3.91)	1.05 (0.41, 2.69)	0.72 (0.31, 1.68)	1.06 (0.76, 1.47)
**Convenience Stores**	1.34 (0.52, 3.47)	1.46 (0.52, 4.13)	1.66 (0.64, 4.33)	1.35 (0.49, 3.70)	1.18 (0.48, 2.92)	1.12 (0.44, 2.86)

All models adjusted for education, income, race, employment, age, and gender.

Sweetened beverage is never consuming a sweetened beverage during the week.

Fruit&veg recommendation ref is eating vegetable, green leafy and fruit less than 2 times per day.

Baked goods and desserts is less than 5 times per week.

Milk is less than once per day.

Cereal is less than once per day.

Red meat is less than 2 times per week.

Frequency of shopping is 0 or never for reference compared to shopping at store type at least once a week.

Bold & * = P≤0.05.

In models assessing the frequency of shopping at certain food venues (Table 4), for every day per week increase in shopping at a supermarket there are higher odds of consuming a sweetened beverage. Yet, for every increase in shopping at supercenter, was associated with lower odds of consuming a sweetened beverage. Lastly, for every day per week increase in shopping at a specialty market or farmers’ market was associated with higher odds of consuming at least two servings of fruits and vegetables relative to consuming less than two servings.

In models examining the associations between overall NEMS-S Rudd score (total score of availability, price, and quality together (Table 5)) the only significant finding was that those who shopped in a store with high availability of healthy food had lower odds of consuming sweetened beverages compared to those who shop in a supermarket with low availability of healthy food.

**Table 5:** Consumer Food Environment (Food availability, price, and quality within stores) and the odds of dietary intake, Lexington, KY 2011

**Total NEMS-S Rudd Score for Each Store Type (Odds Ratio and 95% Confidence Interval)**						
	**Fruit & Vegetable**	**Sweetened Beverages**	**Milk**	**Red Meat**	**Baked Goods and Sweets**	**Cereal**
**Supermarket Store Availability**	0.95 (0.83, 1.08)	0.65 (0.14, 0.83)*	0.92 (0.72, 1.09)	0.95 (0.38, 1.08)	0.94 (0.38, 2.39)	1.05 (0.93, 1.20)
**Supercenters and Warehouse clubs Availability**	1.22 (0.75, 1.99)	1.03 (0.65, 1.65)	1.26 (0.63, 2.57)	1.09 (0.65, 1.82)	0.61 (0.14, 2.67)	0.97 (0.61, 1.54)
**Specialty Store Availability**	0.91 (0.77, 1.08)	0.96 (0.81, 1.14)	1.25 (1.08, 2.56)	0.96 (0.77, 1.19)	0.83 (0.57, 1.21)	1.18 (0.95, 1.46)

All models adjusted for education, income, race, employment, age, and gender.

Sweetened beverage is never consuming a sweetened beverage during the week.

Fruit &vegetable recommendation ref is eating vegetable, green leafy and fruit less than 2 times per day.

Baked goods and sweets is less than 5 times per week.

Milk is less than once per day.

Cereal is less than once per day.

Red meat is less than 2 times per week.

Supermarket and specialty store categorized as "healthy" has a score of 56 or higher.

Supercenter categorized as "healthy" has a score of 57 or higher.

Bold and * = P≤0.05.

## Discussion

The results of this study overall suggest that the type of store a person chooses to shop in, how often they shop, and the availability of healthy food all are associated with certain dietary variables. Two key findings suggest that shopping at a supermarket frequently is associated with consuming sugar-sweetened beverages. However a more nuanced look at the results suggest that it is not the mere habit of frequently shopping but perhaps more a function of availability of food within the store. Yet, our results highlight the nuance of shopping at a supermarket among a higher socio-economic population. One recent study found that frequently shopping at a supermarket was associated with intake of low-fat foods [48]. However, our results did not support this and rather the converse. Yet, at further inspection the nuance of supermarket shopping and the association with diet reveals that not all supermarkets are created equal. Those who shopped at a supermarket with high availability of healthy food reported lower odds of consuming sugar-sweetened beverages. Overall, it’s not simply the mere act of frequently shopping but also the type of supermarket where individuals shop that may influence intake and weight status [21].

This study’s finding that individuals will travel for the type of food venues that meet their needs and or preferences regardless of the healthfulness of their food activity space corroborates previous findings. Previous studies reported that those who participated in the Farmer’s Market Nutrition Program had higher odds of consuming fruits and vegetables [49] compared to those not participating in the program. As those who prefer to purchase fresh produce would more likely seek out farmer’s markets, it is not surprising that those who shopped at farmers’ markets reported higher consumption of fruits and vegetables. Recent studies have reported that type of store is associated with how far people travel for food purchases [50]. Caution is warranted in interpretation of this result, as the sample was socioeconomically advantaged, and if they had chosen to shop somewhere else, fruit and vegetable intake may still be similar. Since the counterfactual cannot be tested future larger scale studies assessing the role of farmers’ market location and temporal nature of this establishment are warranted.

This study has several limitations. A large and severe limitation is the type of sampling procedure used, which produced a homogenous study sample in terms of socio-economics. The sample which participated in this study was older and higher socio-economic status relative to the general population within the city. The sample was not representative of the city or of the general United States. This homogeneity limited the variability in our sample, and thus power to detect significant associations. Although every attempt was made to recruit a diverse sample, response rates were low and therefore selection bias may be an issue in our analyses. Perhaps results simply reflect the food shopping habits of a high SES study population, who generally may have better transportation and a healthier food activity space. Socio-economically advantaged consumers may have more resources to select healthier food venues than less socio-economically advantaged consumers. Although our analyses adjusted for socio-economic characteristics, findings are limited in generalizing to larger populations and low-income groups. Additionally, household composition such as number of children in household was not collected and thus might be cofounder in the relationship between diet and neighborhood food environment. Coupled with an older higher socio-economic sample was that the sample was small and thus underpowered to detect associations. Therefore results may be spurious and thus future studies are needed among a larger more representative sample before conclusions can be made. However, the novel approach of using GPS to define travel patterns and the exposure to food venues within the travel patterns sheds light on different methods for measuring the relationship between the individual and their neighborhood. Yet, a key concern is the use of 3-day travel logs to define usual travel. To date there are no studies validating the use of GPS against a gold standard to measure usual travel. As such 3-day record may be insufficient to capture usual travel. However, in a recent review of of spatial methods most studies used a 3-day pattern [51]. Additionally, the GPS data logger did not capture more complex travel behavior such as tours and trip chains [39,52]. Trip chains or tours often involve such complex travel behavior as traveling from home to a coffee shop, dry cleaner, grocery store, and ending at the office. Future studies need to capture and examine travel patterns for purchasing food to improve our understanding of how individual travel patterns influence food venue choice.

This manuscript did not capture the complex behavior of food purchasing clustering habits. Individuals who purchase large amounts of fruits and vegetables most likely purchase lower calorie, nutrient dense items at the same time. This type of dietary pattern was not captured and be more reflective of a healthy lifestyle rather than focusing on one food group. Lastly, the results suggest that although the ratio of healthy to unhealthy food venues within travel pattern may not be relevant the availability of food within those stores and where individuals choose to shop may influence dietary intake. The limitation of the RFEI measure is that not all supermarkets have the same availability and prices. Thus when grouping supermarkets into one group the result is that all supermarkets are being treated as though they are equal. However, based on the small sample stratification within supermarkets was not feasible and thus the RFEI was used for all models.

## Conclusions

Our findings do not support the notion that living closer to a particular food venue is associated with shopping at that venue, a frequently made assumption that may over-simplify the interdependence between individuals and their environments [53]. More work should be done among residents with varying SES and geographic contexts. As those who shopped more at specialty grocers and farmers’ market were more likely to consume fruits and vegetables. Longitudinal studies should be conducted to examine whether it is that those who like to consume fruits and vegetables shop at such venues, or whether the venues themselves encourage greater consumption of fruits and vegetables. Additionally, a complex relationship between frequently shopping at a supermarket was associated with sugar-sweetened beverage consumption yet, shopping at a supermarket with high availability was associated with lower odds. This relationship suggests that it is not merely the presence of a supermarket that influences behavior but the availability within that store. Thus, interventions and policies aimed at improving intake while also decreasing intake of higher calorie foods need to consider food venue choice but also availability within stores.

## Competing interests

The authors report no financial or competing interests.

## Authors’ contributions

SL assisted with data collection. KM assisted with data collection. SJP assisted with writing and revising of the manuscript. AG conducted data analysis, interpretation of the data, writing, and decision of publication. All authors read and approved the final manuscript.
